# A point system to predict the future risk of obesity in 10-year-old children

**DOI:** 10.1265/ehpm.22-00270

**Published:** 2023-04-21

**Authors:** Risa Sonoda, Mikiko Tokiya, Kenichi Touri, Yuichi Tanomura, Kimihiro Yada, Yayoi Funakoshi, Isao Saito

**Affiliations:** 1 Department of Public Health and Epidemiology, Faculty of Medicine, Oita University, 1-1 Idaigaoka, Hasama-machi, Yufu City, Oita 879-5593, Japan; 2 Department of Social and Environmental Medicine, Faculty of Medicine, Saga University, 5-1-1 Nabeshima, Saga City, Saga 840-8501, Japan; 3 Beppu City Medical Association Community Health Center, 15-33 Nishinoguchi-machi, Beppu City, Oita 874-0931, Japan; 4 ICT & Community Medical Cooperation Office and Community Health Center, 15-33 Nishinoguchi-machi, Beppu City, Oita 874-0931, Japan; 5 Yada Kodomo Clinic, 4-5-4 Ishigakihigashi, Beppu City, Oita 874-0919, Japan

**Keywords:** Epidemiology, Obesity, Pediatric, POW, Prediction model, Prevention

## Abstract

**Background:**

A 4-year longitudinal study was conducted to develop a model and a point system for predicting childhood obesity.

**Methods:**

This study included 1,504 Japanese 10-year-old children who underwent health check-ups between 2011 and 2015. Multivariable logistic regression analysis was conducted using the explanatory variables overweight and lifestyle. Obesity was defined as percentage overweight (POW) ≥ 20% calculated by the following equation: (actual weight − standard weight by height and sex)/standard weight by height and sex × 100 (%). The model was validated using the Hosmer-Lemeshow test on 10-year-olds.

**Results:**

Our prediction model for development of childhood obesity was based on seven binary variables: sex, lack of sleep, ≥2-h use of television/ games/ smartphone, hypertension, dyslipidemia, hepatic dysfunction, and being overweight. The area under the curve of the receiver operating characteristic curve was 0.803 (95% confidence interval, 0.740 to 0.866). When validated in non-obese children (n = 415), there was no significant difference between actual and predicted numbers of children with obesity (Hosmer-Lemeshow chi-square = 7.90, *p* = 0.18).

**Conclusions:**

The validated prediction model and point score for obesity development were shown to be useful tools for predicting the future 4-year risk of developing obesity among 10 years-old children. The point system may be useful for reducing the occurrence of childhood obesity and promoting better health.

**Supplementary information:**

The online version contains supplementary material available at https://doi.org/10.1265/ehpm.22-00270.

## Introduction

Obesity in childhood has both short and long-term effects on health [1]; therefore, measures ought to be taken to reduce the risk of obesity in children. Short-term effects of childhood obesity include physical disorders such as hypertension, impaired glucose metabolism, dyslipidemia, nonalcoholic fatty liver disease, hyperuricemia, and premature atherosclerosis, as well as psychological problems such as low self-esteem and depression [2–9]. Long-term effects of childhood obesity include an increased risk of developing cardiovascular diseases, diabetes, cancer, and musculoskeletal diseases [10–12]. Therefore, measures to curb childhood obesity are necessary at a time when healthy lifestyle habits can and should be encouraged [13].

In some instances, childhood obesity can be attributed to genetic disorders such as Prader-Willi syndrome, hormonal disorders including Cushing’s syndrome that contribute to weight gain, and medications such as corticosteroids. However, 90% of childhood obesity in Japan is attributed to a complex interaction between genetically determined physical characteristics, appetite, nutrient intake, physical activity, energy expenditure, and environmental factors, and there is considerable potential to reduce the risk of obesity through lifestyle improvements [14, 15].

Current reports in Japan indicate the need to prioritize the treatment of childhood obesity, and each municipality conducts health checkups for children to reduce their risk of succumbing to lifestyle-related diseases [14]. Lifestyle-related diseases such as hypertension, obesity, diabetes, and hyperlipidemia can develop and progress among children when, by environmental association, poor parental or caregiver feeding behaviors and lifestyle habits adversely impact their health [14].

Implementing improvements requires the child’s and caregiver’s cooperation. A point system for predicting the onset of disease was developed based on the Framingham Heart Study and has been used to predict and reduce the risk of cardiovascular disease [16]. Some models have been developed to predict the onset of obesity in children [17, 18]. In a Japanese population-based birth cohort from the Hokkaido Study on Environment and Children’s Health of 6,846 mother-child pairs, a childhood obesity risk score was constructed based on genetic predictors identified in pregnant women and 1-year-old infants [18]. However, none of the previous studies evaluated children’s lifestyle and clinical characteristics in Japan.

Since 2011, health checkups have been conducted in children ages 10 and 14 years registered as living in Beppu City. To date, longitudinal data (from 2011 to 2020) are available regarding lifestyle, family history of disease, and blood test results among 10- and 14-year-olds. We investigated the epidemiological and clinical characteristics associated with the development of obesity in Japanese children. In addition, we evaluated whether these characteristics could be used to determine a risk score to predict which children may need support and intervention. The purpose of this study was to construct a prediction model of obesity that also considers lifestyle-related factors.

## Methods

### Study population

**Fig. 1:**
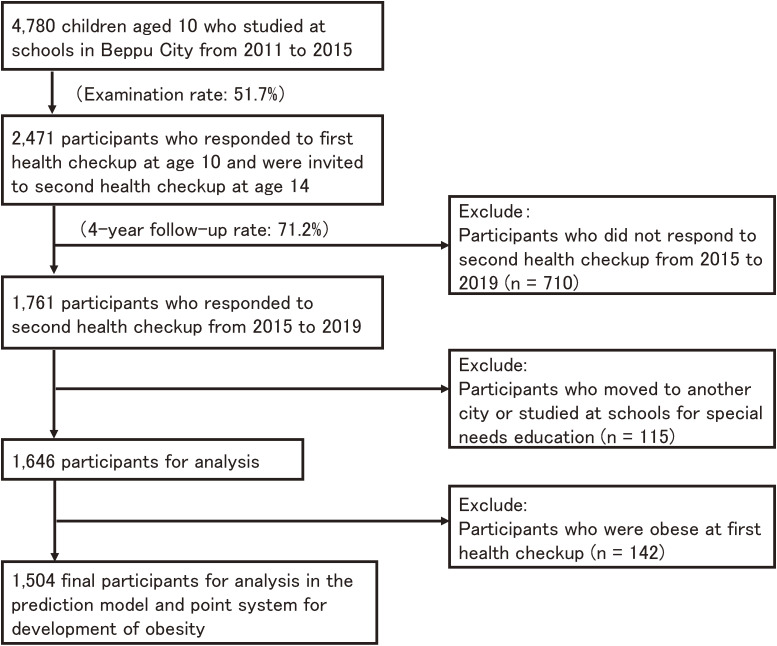
Flow diagram of study participants

This study was conducted using data from the Health Checkup for Prevention of Lifestyle-related Diseases for Children and Students initiative in Beppu City from 2011 to 2020. Study participants were children who received health checkup examinations at both 10 and 14 years of age. There were a total of 4,780 10-year-old students from 2011 to 2015 in elementary schools in Beppu City. Of these, 2,471 participants responded to the first examination; the examination rate was 51.7%. Four years later, 1,761 of those initially examined participated in the second examination as 14-year-olds from 2015 to 2019; the follow-up rate was 71.2%. We excluded 115 participants who moved to another city or studied at schools for special needs education. In all, 1,646 10-year-old boys and girls were included in the analysis. After excluding obese children (n = 142), a total of 1,504 children were included in the prediction model and point system for development of obesity (Fig. 1). To validate the prediction model and point system, we defined another population comprised of 415 children who were screened in both 2016 (at age 10) and 2020 (at age 14), and who were not obese at age 10 (Supplementary Figure 1).

This analysis included data obtained from the Beppu City Health Service. This data was collected for analysis for the prevention of lifestyle-related diseases in children and does not contain personally identifiable information. Study participants were allowed to opt-out of the study using the Oita University and Beppu City websites. This study was approved by the Ethics Committee of Oita University (Approval No. 1790).

### Questionnaires

A self-administered questionnaire was obtained from parents about their child’s school, physical condition, diet, frequency of exercise, family history of diabetes, heart disease, stroke, kidney disease, hypertension, hyperlipidemia, sleep duration, and any illnesses being treated.

A family history of a diseases of interest was defined as having a second-degree relative who was diagnosed with that disease. Lack of exercise was defined as exercising less than twice a week outside of school hours. Lack of sleep was defined as <9 h of sleep for 10-year-olds, per the National Sleep Foundation’s sleep time duration recommendations [19].

### Measurements

Anthropometric (height and weight) and sitting blood pressure measurements were collected at each medical institution. We followed the Japanese Society of Hypertension Guidelines for the Management of Hypertension (JSH2009) [20] for blood pressure measurement in this study. After children were allowed to rest in a quiet environment, measurements were taken three times using an automatic or manual blood pressure cuff for children, and the value of the third of three consecutive measurements was adopted in accordance with the guidelines. Venous blood samples (7 ml) were drawn at each institution between 9:00 to 18:00, and the time that elapsed since the subject’s last meal was documented. Various blood biochemical tests were performed for high-density lipoprotein cholesterol (HDL-C), low-density lipoprotein cholesterol (LDL-C), triglycerides (TG), glutamic pyruvic transaminase (GPT), glutamic oxaloacetic transaminase (GOT), uric acid, and hemoglobin A1c (HbA1c). Levels of GOT, GPT, HDL-C, LDL-C, TG, and uric acid were measured using an automated analyzer (Beckman Coulter Co., Ltd., Tokyo, Japan) and Japan Society of Clinical Chemistry (JSCC) compatible methodology for GOT and GPT; an enzyme method for TG; selective inhibition methodology for HDL-C and LDL-C; and a uricase-peroxidase method for uric acid. HbA1c was measured using the ADAMS A1c HA-8170 glycohemoglobin analyzer (Arkray Co., Ltd., Kyoto, Japan) based on high-performance liquid chromatography mass spectrometry, electrophoresis, or an immunoassay.

Hypertension was defined as systolic blood pressure ≥ 130 mmHg or diastolic blood pressure ≥ 80 mmHg [21]. Hepatic dysfunction was defined as GPT > GOT and GPT ≥ 25 IU/L according to the criteria for non-alcoholic fatty liver disease from the guidelines for childhood obesity [22]. Dyslipidemia and high uric acid levels were defined to align with the Japan Society for the Study of Obesity (JASSO), the guidelines for childhood obesity, namely LDL-C ≥ 140 mg/dL, HDL-C < 40 mg/dL, or TG ≥ 140 mg/dL for blood lipids and a uric acid level ≥ 6 mg/dL regardless of fasting conditions [23]. The threshold for impaired glucose metabolism was set at an HbA1c level of ≥5.8% [23–25]. However, since the criteria for the HbA1c cut-off was revised to match Japan Diabetes Society (JDS) values in 2011 and 2012 and National Glycohemoglobin Standardization Program (NGSP) values in 2013 and later, the HbA1c values recorded in 2011 and 2012 were converted to NGSP values using the formula NGSP(%) = 1.02 × JDS(%) + 0.25%.

### Definition of obesity

Height and weight measurements followed the Manual for Physical Examination of Children used for school physical examinations [26]. The concept of standard weight by sex and height, used to determine obesity, is unique to Japan [14]. Because Asian people are more susceptible to metabolic abnormalities induced by mild obesity than Caucasians, the definition of obesity for adults is lower compared with Caucasians (i.e., body mass index [BMI] ≥ 25 kg/m^2^) [14]. Against this background, mild obesity is considered to have a greater impact on health problems in Japanese as opposed to Caucasian children. The Ministry of Health, Labor and Welfare (MHLW) in Japan categorizes overweight based on percentage of overweight (POW) ≥ 20%, where POW is used to define childhood obesity, and is calculated using the following formula: POW = (actual weight − standard weight by height and sex)/standard weight by height and sex) × 100 (%) [22]. In this study, POW ≥ 20% was also used to define obesity according to the MHLW definition. Overweight was defined as 10–19% of POW.

### Sample size

The minimal number of individuals used for model development N is often considered by the number of events per predictor variable (EPV) [27]. More than 10 EPV is generally required in this situation. The EPV in this study was 9.3 (= 65 outcomes/7 variables) when calculated using outcomes and variables, but an EPV of 5 to 20 is acceptable for model development using a logistic model [28, 29].

### Statistical analysis

The sex-specific proportion of obese and non-obese children was determined for the following binary variables by a response of “Yes” or “No”: family history of hypertension and diabetes; lack of exercise; lack of sleep; ≥2-h use of television/ games/ smartphone; hypertension; dyslipidemia; impaired glucose metabolism; hepatic dysfunction; high uric acid level; and being overweight. All missing data were considered to be a response of “No” for this analysis.

Sex-adjusted logistic regression analysis was conducted for the development of obesity as a dependent variable, and using the obesity-related factors described above as explanatory variables. Among the variables, we selected sex (fundamental) and six other variables with significance levels of *P* < 0.1 in the sex-adjusted model. The Pearson correlation coefficient was used to measure the strength of a linear association between BMI and POW (r = 0.95 for both 10-year-old boys and girls). Multivariable logistic regression models were adopted to estimate odds ratios (OR) and 95% confidence intervals (CI) with the variables we selected for obesity to create the prediction model. As a first step, we explored variables that could predict future obesity in a sex-adjusted model and used them to create multivariate-adjusted models with and without overweight (Models 1 and 2, respectively). Since the overweight factor was a major risk predictor of future obesity, we compared two such models to see its impact. The correlation coefficient was less than 0.16, and we confirmed that there was no collinearity among the variables in models 1 and 2. The weighting of the points was based on the partial regression coefficients obtained in Model 2, with the point for the partial regression coefficient for dyslipidemia set at 1 and weighted according to how many times the partial regression coefficient for each variable was greater than the criterion [16]. The fitness of the models was evaluated using receiver operating characteristic (ROC) curves by calculating the area under the curve (AUC).

To validate the prediction equation, we analyzed data of 415 non-obese children (214 boys and 201 girls) in Beppu City who were examined in both 2016 and 2020 (as 10- and then 14-year-olds, respectively). Validation was done with a multivariate logistic model for development of obesity at age 14 by using partial regression coefficients provided in Model 2. To test the difference between the actual and predicted numbers of subjects with obesity, the Hosmer-Lemeshow test was performed. In addition, the calibration plots were created by using a locally weighted scatterplot smoothing plot with predicted probabilities on the x-axis and observed probabilities on the y-axis evaluated by Spiegelhalter’s z-test statistics [30, 31].

All analyses were done using SAS ver 9.4 (SAS Institute Inc., Cary, NC, USA).

## Results

From 2011 to 2020, health examinations were performed for 826 (50.2%) boys and 820 (49.8%) girls, at 10 years of age. Among them, 81 boys and 61 girls were classified as obese. First, we compared cross-sectional characteristics of the 10-year-old subjects with and without obesity by sex (Table 1). The following factors were found to be associated with obesity in both boys and girls: ≥2-h use of television/ games/ smartphone, hypertension, dyslipidemia, impaired glucose metabolism, hepatic dysfunction, high uric acid level, and being overweight.

**Table 1:** Baseline characteristics of the study population (n = 1,646)

**Variables^b^**	**Boys, n = 826**		***p***	**Girls, n = 820**		***p***
	
	**Obese, n = 81**	**Non-obese, n = 745**		**Obese, n = 61**	**Non-obese, n = 759**	
Family history						
Hypertension	43 (53.1)	348 (46.7)	0.275	32 (52.5)	362 (47.7)	0.474
Diabetes	29 (35.8)	221 (29.7)	0.254	29 (47.5)	241 (31.8)	0.012
Lack of exercise	8 (9.9)	75 (10.1)	0.957	5 (8.2)	50 (6.6)	0.629
Lack of sleep	25 (30.9)	170 (22.8)	0.105	22 (36.1)	181 (23.9)	0.033
≥2-h use of television/ games/ smartphone	39 (48.2)	279 (37.5)	0.060	30 (49.2)	233 (30.7)	0.003
Hypertension	8 (9.9)	25 (3.4)	0.011^a^	7 (11.5)	25 (3.3)	0.007^a^
Dyslipidemia	33 (40.7)	113 (15.2)	<0.001	20 (32.8)	120 (15.8)	0.001
Impaired glucose metabolism	3 (3.7)	8 (1.1)	0.084^a^	3 (4.9)	8 (1.1)	0.042^a^
Hepatic dysfunction	26 (32.1)	35 (4.7)	<0.001	9 (14.8)	20 (2.6)	<0.001^a^
High uric acid level	12 (14.8)	20 (2.7)	<0.001^a^	11 (18.0)	19 (2.5)	<0.001^a^
Overweight	81 (100)	88 (11.8)	<0.001	61 (100)	93 (12.3)	<0.001

All values shown are n (%).

P values were calculated by the chi-square test.

^a^ Fisher’s exact test.

^b^ Definition: family history, having a second-degree relative who was diagnosed with that disease; lack of exercise, exercising less than twice a week outside of school hours; lack of sleep, <9 h of sleep for 10-year-olds; ≥2-h use of television/ games/ smartphone, ≥2-h use of television, games, or smartphone after school; hypertension, systolic blood pressure ≥ 130 mmHg or diastolic blood pressure ≥ 80 mmHg; dyslipidemia, low-density lipoprotein cholesterol ≥ 140 mg/dL, high-density lipoprotein cholesterol < 40 mg/dL, or triglycerides ≥ 140 mg/dL; impaired glucose metabolism, hemoglobin A1c ≥ 5.8%; hepatic dysfunction, glutamic pyruvic transaminase (GPT) > glutamic oxaloacetic transaminase and GPT ≥ 25 IU/L; high uric acid level, serum uric acid ≥ 6 mg/dL; and overweight, 10–19% of the percentage overweight.

Among the 1,504 subjects who were not obese at 10 years of age, 65 developed obesity four years later. The cumulative incidence of obesity over four years was 4.0% (95% CI: 3.0 to 5.0) in our population.

Factors associated with the development of obesity among 1,504 non-obese 10-year-old children are shown (Table 2). There was a trend toward higher ORs for obesity in lack of sleep; ≥2-h use of television/ games/ smartphone; dyslipidemia; hepatic dysfunction; and being overweight. Hypertension was borderline significant. Being overweight was strongly associated with obesity (OR = 12.7, 95% CI: 7.56 to 21.5).

**Table 2:** Sex-adjusted ORs for development of obesity 4 years later among non-obese 10-year-old children (n = 1,504)

**Factors in 10-year-olds^a^**	**Obesity in 14-year-olds**		

	**Sex-adjusted OR**	**95% Cl**	***p***
Girl	1.08	0.66 to 1.78	0.761
Family history			
Hypertension	1.32	0.80 to 2.17	0.276
Diabetes	1.25	0.74 to 2.10	0.409
Lack of exercise	0.92	0.36 to 2.35	0.868
Lack of sleep	1.72	1.02 to 2.93	0.043
≥2-h use of television/ games/ smartphone	1.72	1.04 to 2.83	0.035
Hypertension	2.58	0.99 to 6.74	0.053
Dyslipidemia	2.00	1.13 to 3.55	0.017
Impaired glucose metabolism	3.23	0.72 to 14.5	0.126
Hepatic dysfunction	3.56	1.54 to 8.24	0.003
High uric acid level	1.21	0.28 to 5.11	0.801
Overweight	12.7	7.56 to 21.5	<0.001

^a^ Definition: family history, having a second-degree relative who was diagnosed with that disease; lack of exercise, exercising less than twice a week outside of school hours; lack of sleep, <9 h of sleep for 10-year-olds; ≥2-h use of television/ games/ smartphone, ≥2-h use of television, games, or smartphone after school; hypertension, systolic blood pressure ≥ 130 mmHg or diastolic blood pressure ≥ 80 mmHg; dyslipidemia, low-density lipoprotein cholesterol ≥ 140 mg/dL, high-density lipoprotein cholesterol < 40 mg/dL, or triglycerides ≥ 140 mg/dL; impaired glucose metabolism, hemoglobin A1c ≥ 5.8%; hepatic dysfunction, glutamic pyruvic transaminase (GPT) > glutamic oxaloacetic transaminase and GPT ≥ 25 IU/L; high uric acid level, serum uric acid ≥ 6 mg/dL; and overweight, 10–19% of the percentage overweight.

Cl: confidence interval; OR: odds ratio.

**Fig. 2:**
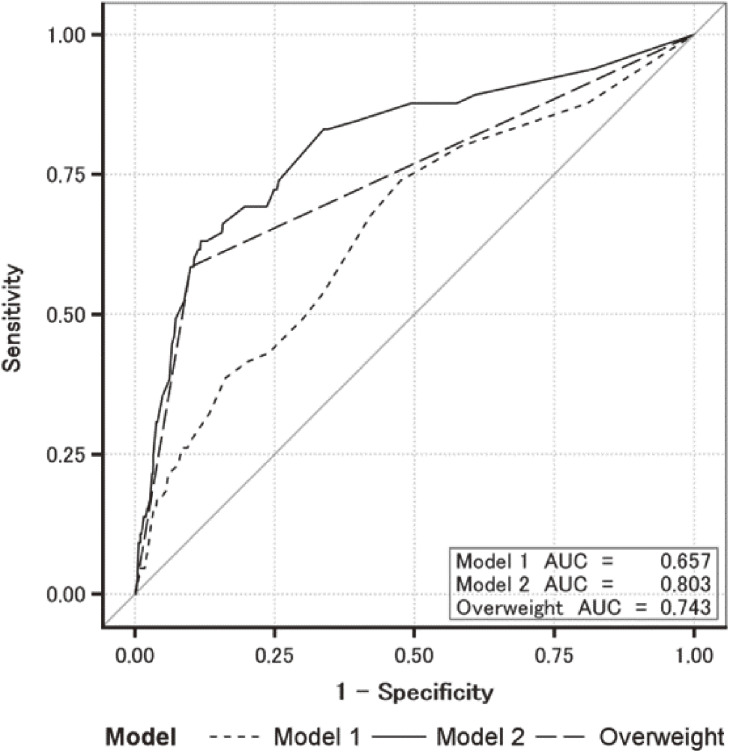
Receiver operator characteristic curves for two prediction models of obesity (n = 1,504, see Table 3)

We created two models, one without overweight as a factor (Model 1) and one with overweight as a factor (Model 2), for the prediction of obesity using the variables shown in Table 3. Sex was included as a fundamental variable. The AUC was 0.657 (95% CI: 0.585 to 0.728) in Model 1 and 0.803 (95% CI: 0.740 to 0.866) in Model 2 (Fig. 2). AUC for the overweight model was calculated separately (AUC = 0.743, 95% CI: 0.682 to 0.804). The AUC when overweight was included in Model 2 was much higher than when overweight was excluded (p < 0.001).

**Table 3:** Multivariable adjusted ORs for development of obesity 4 years later among non-obese 10-year-old children (n = 1,504)

**Variables^a^**	**Model 1**			**Model 2**		
	
	**OR**	**95% Cl**	***p***	**OR**	**95% Cl**	***p***
Girl	1.14	0.69 to 1.89	0.612	1.07	0.63 to 1.82	0.806
Lack of sleep	1.74	1.02 to 2.98	0.042	1.48	0.84 to 2.60	0.178
≥2-h use of television/ games/ smartphone	1.65	0.99 to 2.73	0.055	1.56	0.91 to 2.66	0.103
Hypertension	2.88	1.09 to 7.61	0.033	1.94	0.68 to 5.52	0.217
Dyslipidemia	1.95	1.09 to 3.49	0.025	1.36	0.73 to 2.53	0.335
Hepatic dysfunction	3.30	1.40 to 7.76	0.006	1.58	0.63 to 3.99	0.334
Overweight	—			10.9	6.33 to 18.8	<0.001

Intercept	−3.783			−4.264		
Area under the curve (95% CI)	0.657 (0.585 to 0.728)			0.803 (0.740 to 0.866)		

ORs were adjusted for all variables included in the table.

^a^ Definition: lack of sleep, <9 h of sleep for 10-year-olds; ≥2-h use of television/ games/ smartphone, ≥2-h use of television, games, or smartphone after school; hypertension, systolic blood pressure ≥ 130 mmHg or diastolic blood pressure ≥ 80 mmHg; dyslipidemia, low-density lipoprotein cholesterol ≥ 140 mg/dL, high-density lipoprotein cholesterol < 40 mg/dL, or triglycerides ≥ 140 mg/dL; hepatic dysfunction, glutamic pyruvic transaminase (GPT) > glutamic oxaloacetic transaminase and GPT ≥ 25 IU/L; and overweight, 10–19% of the percentage overweight.

CI: confidence interval; OR: odds ratio.

According to the parameters of each explanatory factor in our models, weighted points were determined. The points for each factor, βi (Wij-Wi_REF_) divided by B (0.3064) ranged from 0 to 8, resulting in a total of 15 points (Table 4). Table 5 shows the probability of being obese corresponding to the point score.

**Table 4:** Point scores of variables to predict obesity

**Variables^a^**	**Reference ** **value** **(W_ij_)**	**βi**	**βi(W_ij_-W_iREF_)**	**Points_ij_ = ** **βi(W_ij_-W_iREF_)/B**
Sex		0.0666		
Boys	0 = W_1REF_		0	0
Girls	1		0.0666	0
Lack of sleep		0.3895		
No	0 = W_2REF_		0	0
Yes	1		0.3895	1
≥2-h use of TV/ games/ smartphone		0.4434		
No	0 = W_3REF_		0	0
Yes	1		0.4434	1
Hypertension		0.6605		
No	0 = W_4REF_		0	0
Yes	1		0.6605	2
Dyslipidemia		0.3064		
No	0 = W_5REF_		0	0
Yes	1		0.3064	1
Hepatic dysfunction		0.4569		
No	0 = W_6REF_		0	0
Yes	1		0.4569	2
Overweight		2.3886		
No	0 = W_7REF_		0	0
Yes	1		2.3886	8

β (0.3064) was defined as dyslipidemia as a constant in the point system.

^a^ Definition: lack of sleep, <9 h of sleep for 10-year-olds; ≥2-h use of television/ games/ smartphone, ≥2-h use of television, games, or smartphone after school; hypertension, systolic blood pressure ≥ 130 mmHg or diastolic blood pressure ≥ 80 mmHg; dyslipidemia, low-density lipoprotein cholesterol ≥ 140 mg/dL, high-density lipoprotein cholesterol < 40 mg/dL, or triglycerides ≥ 140 mg/dL; hepatic dysfunction, glutamic pyruvic transaminase (GPT) > glutamic oxaloacetic transaminase and GPT ≥ 25 IU/L; and overweight, 10–19% of the percentage overweight.

**Table 5:** Point system for prediction of obesity 4 years later among non-obese children (n = 1,504)

**Point total^a^**	**Estimate of risk**		

	**a^b^**	**b^c^**	**(%)**
0	−4.2640	0.0139	1.4
1	−3.9576	0.0188	1.9
2	−3.6512	0.0253	2.5
3	−3.3448	0.0341	3.4
4	−3.0384	0.0457	4.6
5	−2.7320	0.0611	6.1
6	−2.4256	0.0812	8.1
7	−2.1192	0.1072	10.7
8	−1.8128	0.1403	14.0
9	−1.5064	0.1815	18.1
10	−1.2000	0.2315	23.1
11	−0.8936	0.2904	29.0
12	−0.5872	0.3573	35.7
13	−0.2808	0.4303	43.0
14	0.0256	0.5064	50.6
15	0.3320	0.5822	58.2

^a^ Point total (0) is the intercept in Model 2.

^b^ Calculated by the following equation: ΣβiXi = −4.2640 + 0.3064 (constant) × point total.

^c^ b = 1/(1 + e^−a^).

**Fig. 3:**
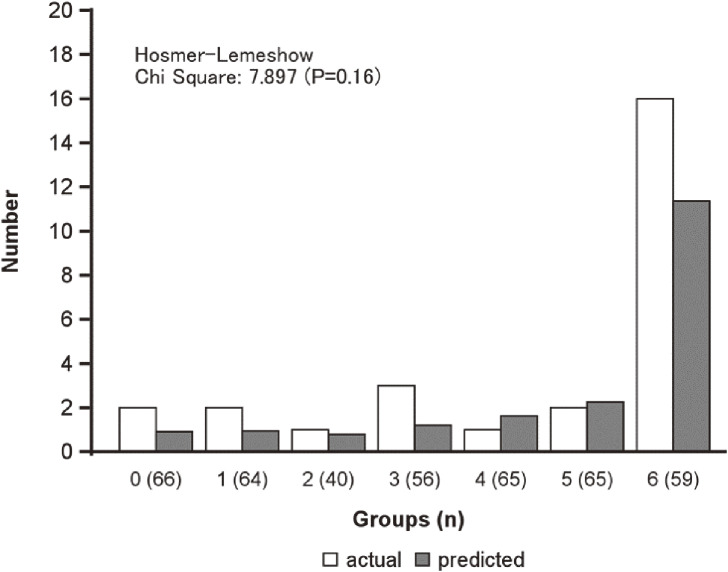
Actual and predicted numbers of subjects with obesity at age 14 by probability values (n = 415)

The validity study was conducted on 415 at 10 years of age who were not obese. The characteristics of the population are presented (Supplementary Table 1). The population was homogeneous with the population for which the predictive model was built. 27 (6.5%) developed obesity four years later. We defined seven groups according to the probability of developing obesity, as calculated by the prediction model (n = 415). The actual and predicted numbers of subjects with obesity were compared among the groups (Fig. 3). There was no significant difference between the two groups using the Hosmer-Lemeshow test (chi-square = 7.897, p = 0.18). Furthermore, calibration plots indicated the good fitness of the predictive model we created (Spiegelhalter’s z-score, −0.56, p = 0.58) (Supplementary Figure 2).

## Discussion

We found that obesity at age 14 can be predicted using the following factors at age 10: sex; lack of sleep; ≥2-h use of television/ games/ smartphone; hypertension; dyslipidemia; hepatic dysfunction; and being overweight. Being overweight at age 10 was strongly associated with future development of obesity. The AUC of the model constructed in this study increased by 0.803 (95% CI: 0.740 to 0.866) when overweight was included as a predictive factor. Validation study indicated that this predictive model is a good fit.

The purpose of this study was to develop a predictive model that included lifestyle factors and overweight status. The goal was to develop a tool that could motivate people at an early age to prevent the development obesity in the future. Sex was included as a factor in the prediction model; however, the point score was zero because the correlation coefficient was small. The remaining six factors selected were useful to estimate the future risk of developing obesity in children using the point system. Overweight had the highest OR for predicting obesity 4 years later in 10-year-olds. The AUC for the model without overweight was 0.657, whereas the inclusion of overweight increased the AUC to 0.803, although the significance associated with the other factors was eliminated. Although other factors were probably mediated by overweight, the inclusion of overweight was deemed more appropriate to increase predictive ability.

The classification of overweight is not yet defined in the guidelines for childhood obesity in Japan [19]. We used a cut-off for overweight of ≥10% POW in the present study, which corresponds to the 80th percentile of the total distribution at age 10. It was expected that the higher the POW value, the greater the risk of developing obesity. However, the addition of lifestyle and blood test conditions also improved the predictive ability.

The predictive model for this study excluded children who were obese at age 10. Of the 142 obese children, 69 (48.9%) were still determined to be obese at age 14. In addition, obese individuals at age 10 had a high frequency of conditions such as hypertension, dyslipidemia, hepatic dysfunction, and high urinary acid levels, suggesting that being obese at age 10 is itself a high-risk individual for future obesity. In other words, this prediction model should be used for non-obese children at age 10, and children who are already obese should receive immediate health guidance regardless of obesity prediction, as they are at high risk for future obesity.

Although number of studies have proposed point systems to predict future childhood obesity risk, most were based on factors at birth or the mother’s pregnancy [32–34]. It was reported that childhood obesity in Japan can be predicted by the mother’s pre-pregnancy BMI, the child’s gender, smoking at the time of pregnancy, the mother’s educational history, and obesity level at 1 year of age [18]. To date, no prediction model in the age range investigated in this study has been developed in Japan. The unique feature of the present study is the development of a prediction model of obesity risk that can be utilized among 10-year-old children, which is an age acceptable for health education. Moreover, this tool can be used to provide educational interventions aimed at obesity prevention among both parents and children.

In this study, lack of sleep was included in the predictive model for the development of obesity in children. Lack of sleep as a predictor of future obesity has been reported in numerous other longitudinal studies [5–7, 35]. A meta-analysis that included intervention studies, showed that reducing the hours of sleep for children was associated with the development of obesity via insulin resistance, unhealthy dietary patterns, and sedentarism [36]. Sleep affects important hippocampus and prefrontal cortex functions, which are necessary for optimizing both physical and mental development, as well as learning [37]. Chronic sleep deficiency perturbs immune homeostasis, inhibits the secretion of growth hormone, and reduces the ability of the body to recover from fatigue [38, 39]. Based on these findings, it is expected that getting the recommended amount of sleep will contribute to healthy development and reduce the risk of future obesity.

Using logistic regression analysis and the prevalence of obesity in our study as the dependent variable to determine future effects of obesity onset within 4 years, some explanatory factors (i.e., family history of hypertension and diabetes, lack of exercise, impaired glucose metabolism, and high uric acid level) proved unpredictive. Follow-up studies reported by Kubo have shown that early interventions (at preschool ages) are necessary to reduce childhood obesity [14]. This notion was inferred given that approximately 30% of the women studied who were overweight at ages 3 and 6 were overweight as adults, and increases in BMI by ages 7 to 8 years were positively correlated with BMI in adulthood [14, 40, 41]. Obesity can also be lifestyle-related during childhood; it may be anticipated that presenting individual point scores during health checkups can lead to improvements that reduce the risk of becoming obese [42–45].

Since high uric acid levels were not associated with obesity at 4 years later among 10-year-old children, we did not include them in our predictive model. A previous study reported that serum uric acid levels increased significantly among adolescents (aged 9.1 to 15.0 years; POW ≥ 20%) who were obese; similarly, serum uric acid levels are associated with obesity in adults [40]. Notably, the direct mechanism underlying the development of cardiovascular disease and type 2 diabetes mellitus with excessive sugar intake involves the unregulated hepatic uptake and metabolism of fructose in the liver, causing hepatic lipid accumulation, dyslipidemia, decreased insulin sensitivity, and increased uric acid levels [41]. Furthermore, in a linear regression analysis examining the changes in BMI and weight in 102 obese patients (mean age, 11 years), resistin/uric acid levels predicted an increase in BMI after 1 year, while only uric acid levels decreased similarly to what was observed in our study [46]. Based on the negative findings, we propose that an elevated uric acid level is not a predictor of obesity per se, but may be a condition resulting from hepatic dysfunction or dyslipidemia. Differences in study design (i.e., cross-sectional or longitudinal) may also be influential in terms of predictiveness. The association between a family history of diabetes and obesity in children is well known [14], and the results of our study are consistent with this in girls. However, similar to uric acid, negative estimates suggest that family history of diabetes may not be an independent predictor of future obesity.

In Japan, annual health checkups in school are conducted under the School Health and Safety Act. Blood tests are not recommended because they are invasive. Apart from this law, many schools conduct health checkups, including blood tests voluntarily for children ages 10 (or 11) and 14 (or 13) to prevent lifestyle-related diseases. The accuracy of our prediction model was found to greatly improve when the results of blood tests were included, suggesting that while blood testing is not necessary it can certainly help to predict the future risk of developing obesity.

Although the main strength of our study is that we created a point score for the development of obesity and presented an estimate of risk based on data obtained longitudinally over 4 years, some study limitations exist. First, this study was conducted using data from previous health examinations. It is not a study designed to create our predictive model. Therefore, the only variables that could be used were those used in previous questionnaires. Study participants were limited to one region of Japan. In addition, the longitudinal analysis was limited to those who had been examined at both 10 and 14 years of age, making selection bias inevitable. Second, the baseline for follow-up of participants was 10 years of age, which may not be early enough. A report of a cohort of Japanese subjects showed that the condition of being overweight in early childhood can continue into adulthood, and that there is a need for early preschool intervention [14]. Third, our calculation of obesity was based on the standard weight by sex and height formula applicable to children living in Japan. Therefore, for global application, it is necessary to use the standard weight by age and height formula unique to the participant’s country. Fourth, we asked parents for information on lifestyles and family history. If parents had concerns about their child being obese when completing the questionnaire, this may have resulted in information bias. In the present study, the questionnaires on physical inactivity and sleep deprivation could not avoid measurement bias. Fifth, we did not have access to a family history of obesity, which might be an important determinant for predicting obesity in child. However, determining the accuracy of such data (i.e., whether or not a family member is obese) would be difficult.

In conclusion, we developed a valid prediction model and point scores for development of obesity among 10-year-old boys and girls. Results of this study suggest that the point system may be useful for reducing the future risk of childhood obesity and promoting better health.

## Supplementary information

**Additional file 1:** **Supplementary Table 1.** Baseline characteristics of the study population for validation (n = 415). **Supplementary Figure 1.** Flow diagram of non-obese study participants used for model validation. **Supplementary Figure 2.** Calibration plot for the validation population (n = 415).

## Declarations

### Availability of data and material

Not available.
