# Supplementary material for: A point system to predict the future risk of obesity in 10-year-old children
Source: Environ Health Prev Med. 2023 Apr 21;28:25. doi: 10.1265/ehpm.22-00270 (PMC10149319; doi:10.1265/ehpm.22-00270)
Supplement: Supplementary file 1 — Additional file 1: Supplementary Table 1. Baseline characteristics of the study population for validation (n = 415). Supplementary Figure 1. Flow diagram of non-obese study participants used for model validation. Supplementary Figure 2. Calibration plot for the validation population (n = 415). [file ehpm-28-025-s001.docx]

**Supplementary Table 1**. Baseline characteristics of the study population for validation (n=415)

|  | Non-obese children in 10-year-olds | |
| --- | --- | --- |
|  | Boys, n=214 | Girls, n=201 |
| Family history |  |  |
| Hypertension | 102 (47.7) | 92 (45.8) |
| Diabetes | 68 (31.8) | 67 (33.3) |
| Lack of exercise | 40 (18.7) | 67 (33.3) |
| Lack of sleep | 53 (24.8) | 43 (21.4) |
| $\geq$2-h use of television/ games/ smartphone | 90 (42.1) | 84 (41.8) |
| Hypertension | 6 (2.8) | 11 (5.5) |
| Dyslipidemia | 43 (20.1) | 45 (22.4) |
| Impaired glucose metabolism | 3 (1.4) | 5 (2.5) |
| Hepatic dysfunction | 13 (6.1) | 5 (2.5) |
| High uric acid level | 2 (0.9) | 3 (1.5) |
| Overweight ^a^ | 27 (12.6) | 28 (13.9) |

All values shown are n (%).

^a^ Overweight was defined as 10-19% of the percentage overweight.

　　
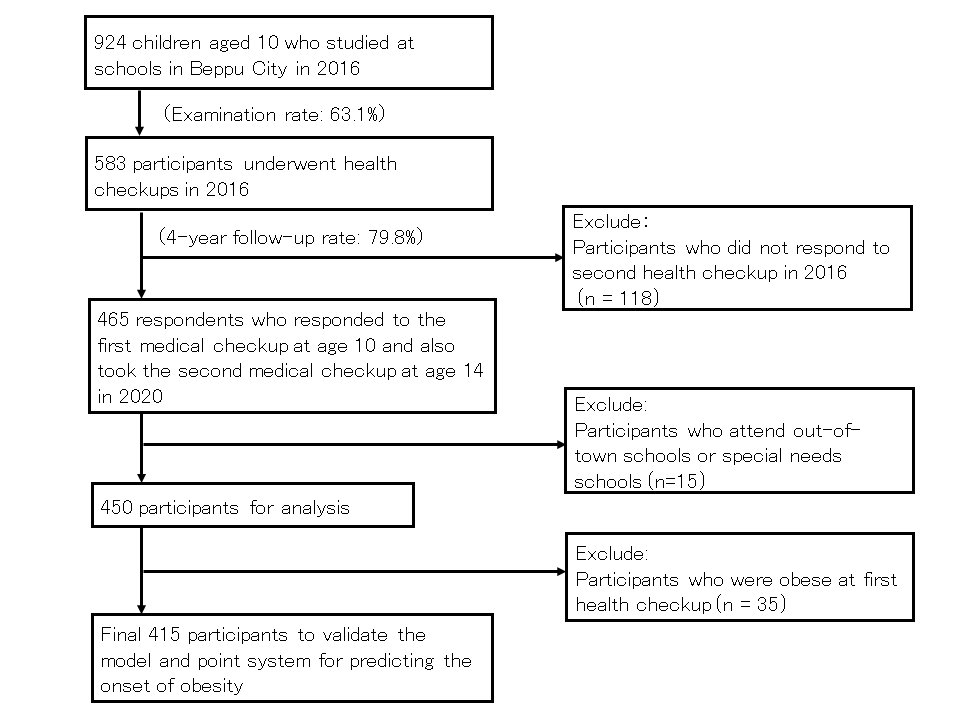


Supplementary Figure 1. Flow diagram of non-obese study participants used for model validation.

　　
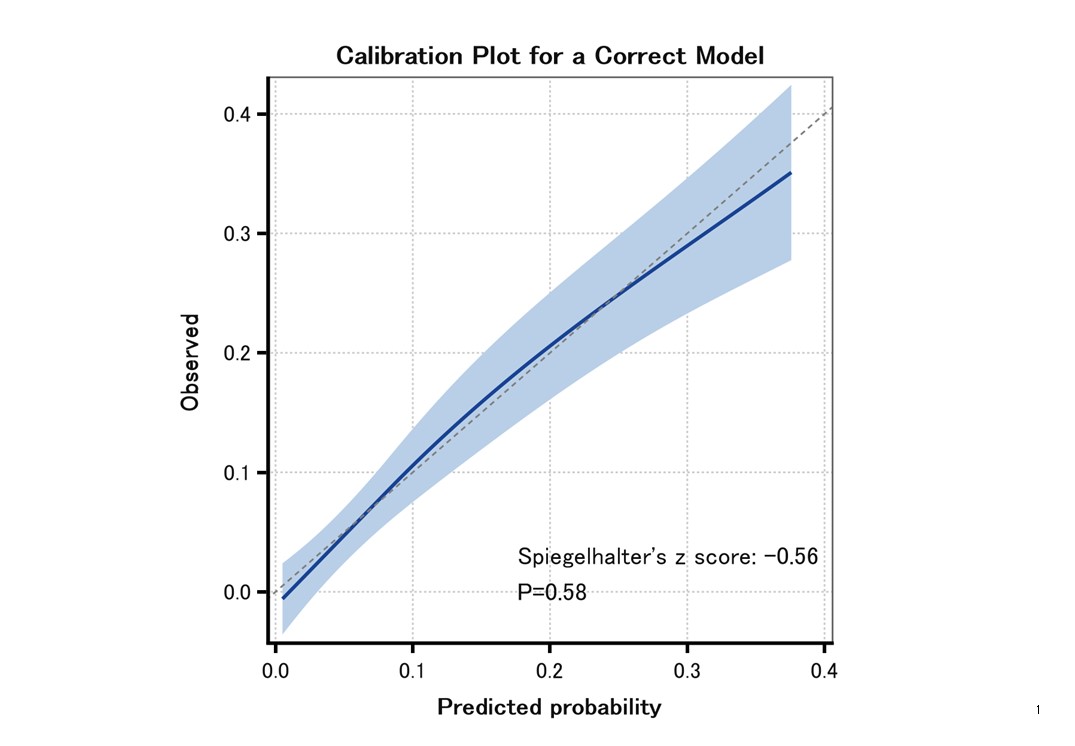


Supplementary Figure 2. Calibration plot for the validation population (n=415).
